# Supplementary material for: SREBP-Dependent Regulation of Lipid Homeostasis Is Required for Progression and Growth of Pancreatic Ductal Adenocarcinoma
Source: Cancer Res Commun. 2024 Sep 27;4(9):2539–52. doi: 10.1158/2767-9764.CRC-24-0120 (PMC11444119; doi:10.1158/2767-9764.CRC-24-0120)
Supplement: Supplementary Figure 1 — FIGURE S1 – Scap is not required for development or function of the mouse pancreas. [file crc-24-0120_supplementary_figure_1_suppsf1.pdf]

Supplementary Figure 1

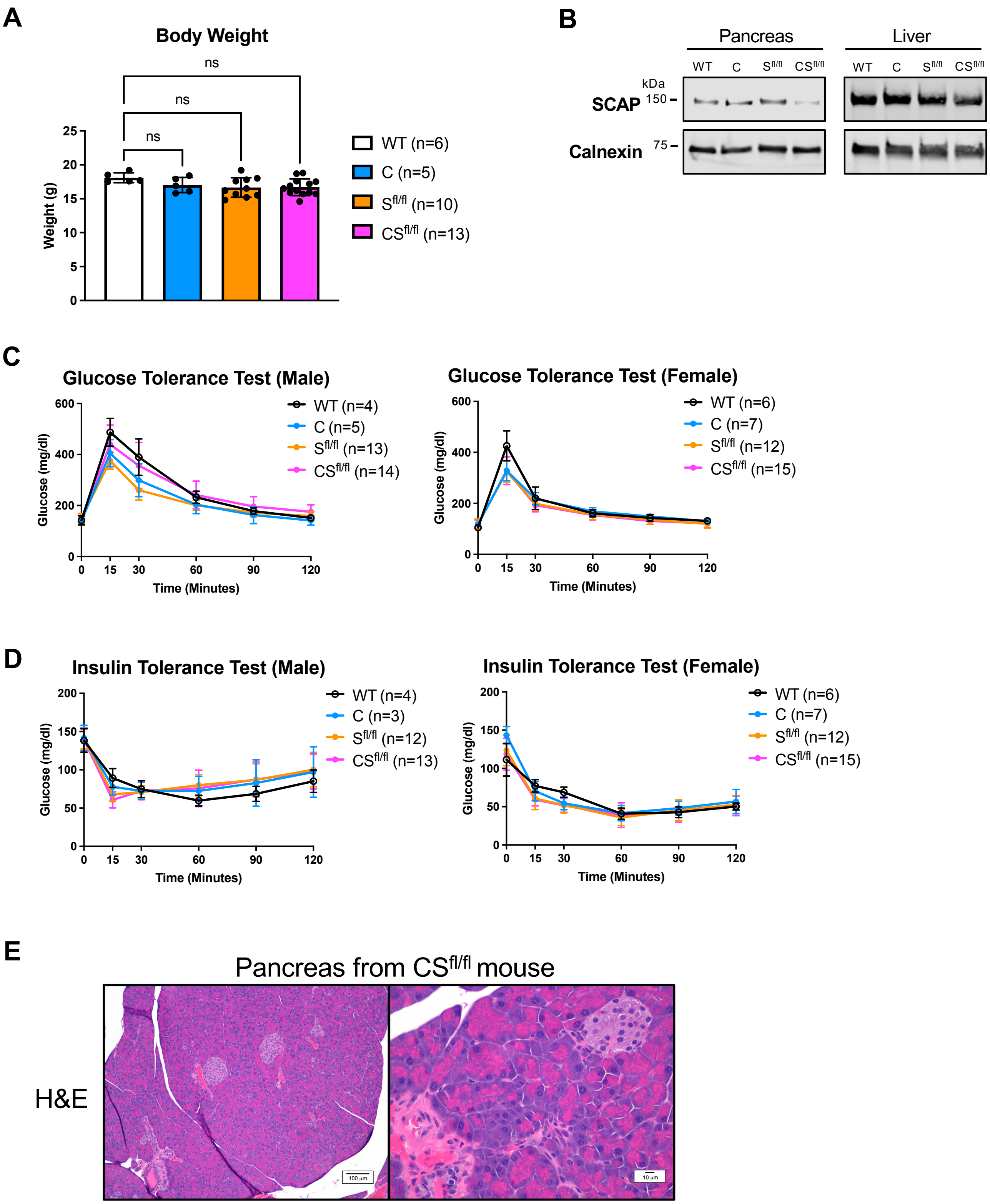

FIGURE S1 – *Scap* is not required for development or function of the mouse pancreas.

A) Body weights of 6-week-old female mice of C57BL/6 wildtype (WT), Pdx1-Cre (C), $Scap^{fl/fl}$ ($S^{fl/fl}$), or Pdx1-Cre $Scap^{fl/fl}$ ($CS^{fl/fl}$) mice. Statistical significance was determined by one-way ANOVA. Error bars denote standard deviation. **B)** Immunoblot analysis of microsomal membranes (40 µg) isolated from mouse pancreas and liver from indicated genotypes (1 mouse per sample). Blots were probed for SCAP and calnexin served as a loading control. **C)** Glucose tolerance tests in 6-week-old male (left graph) and female (right graph) mice with indicated genotypes. Error bar denotes standard deviation. **D**) Insulin tolerance test in 6-week-old male (left graph) and female (right graph) mice with indicated genotypes. Error bar denotes standard deviation. **E)** Representative H&E sections of the pancreas from a $CS^{fl/fl}$ mouse at low magnification (10X, left image) and higher magnification (40X, right image).
