# Supplementary material for: SREBP-Dependent Regulation of Lipid Homeostasis Is Required for Progression and Growth of Pancreatic Ductal Adenocarcinoma
Source: Cancer Res Commun. 2024 Sep 27;4(9):2539–52. doi: 10.1158/2767-9764.CRC-24-0120 (PMC11444119; doi:10.1158/2767-9764.CRC-24-0120)
Supplement: Supplementary Figure 2 — FIGURE S2 – Mouse genotyping methods and survival study data [file crc-24-0120_supplementary_figure_2_suppsf2.pdf]

Supplementary Figure 2

A

Mouse #	Genotype
1	C
2	$CS^{fl/+}$
3	$CS^{fl/fl}$
4	KPC
5	$KPCS^{fl/+}$
6	$KPCS^{fl/fl}$

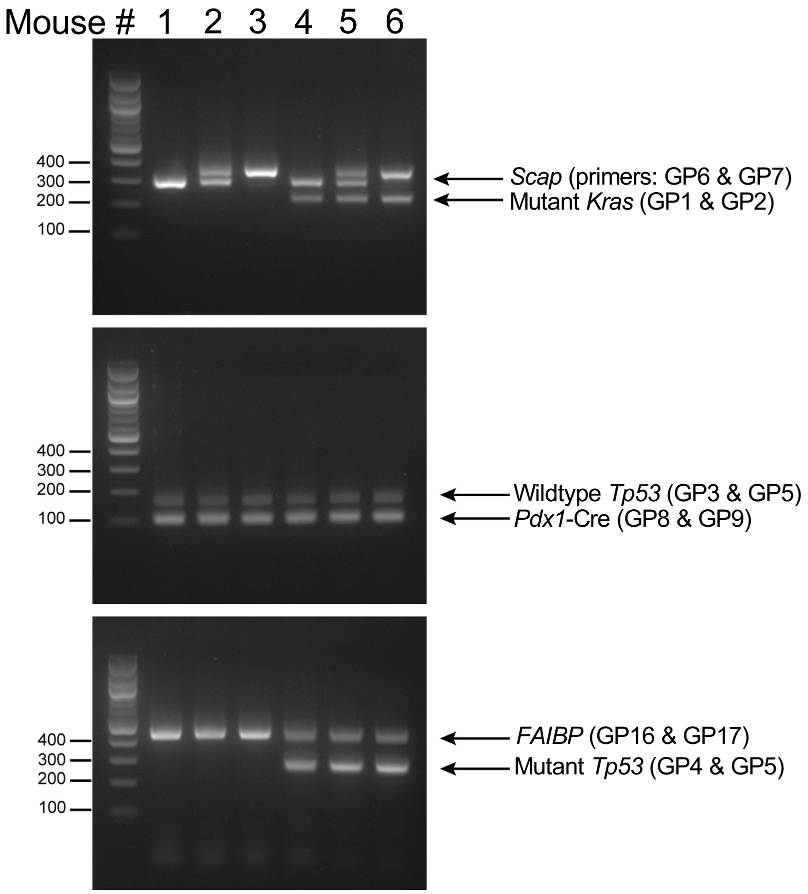

B

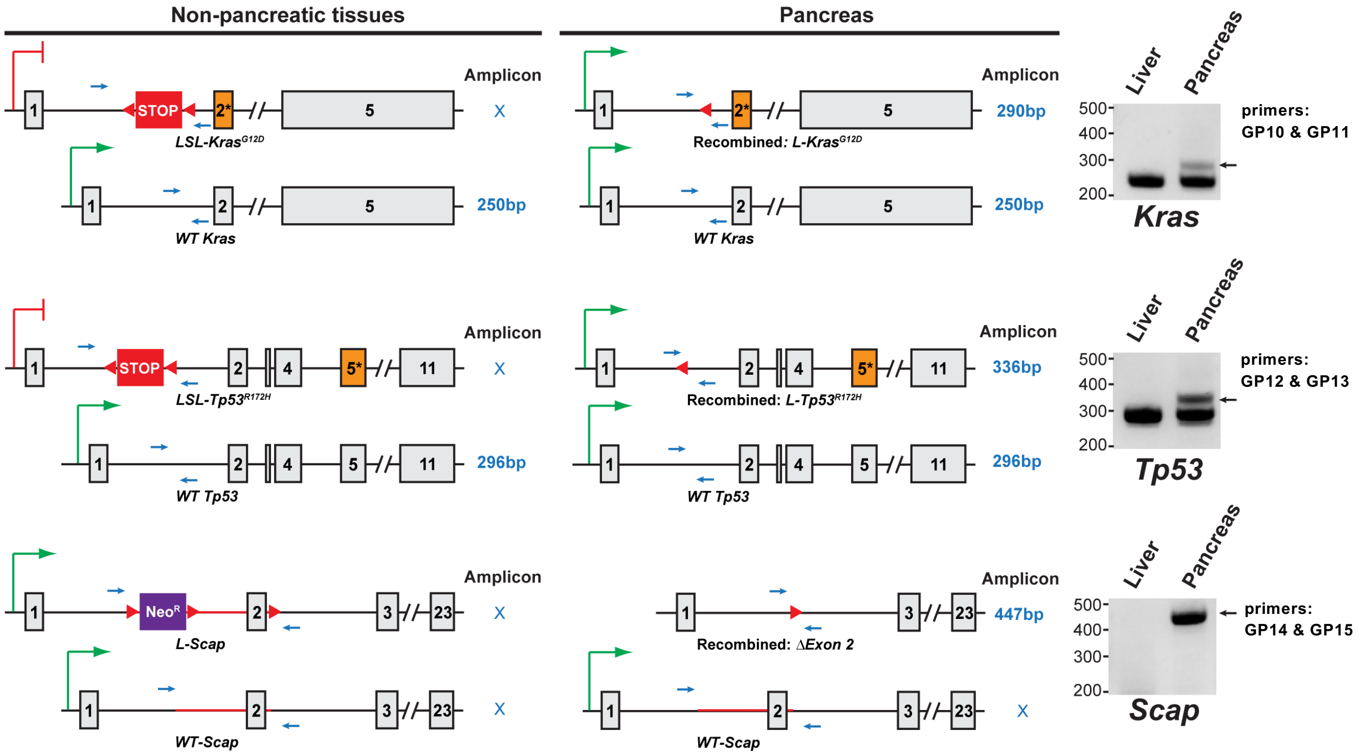

Supplementary Figure 2 continued

C

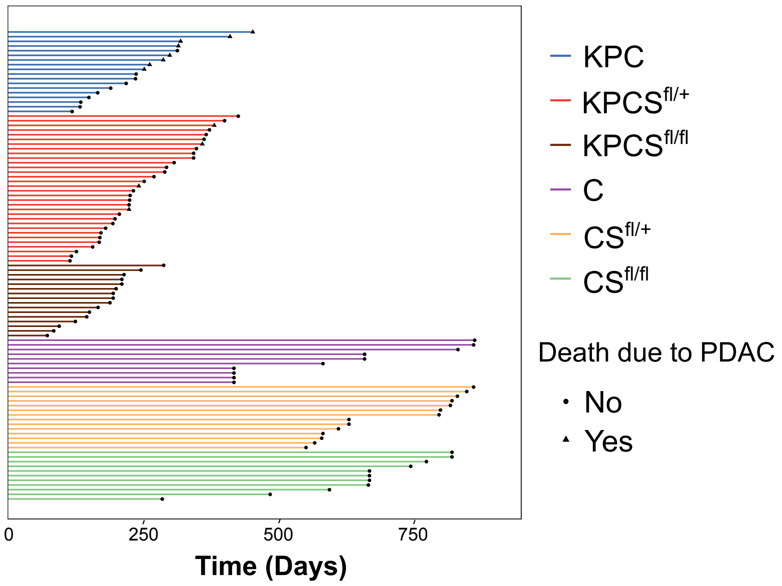

D

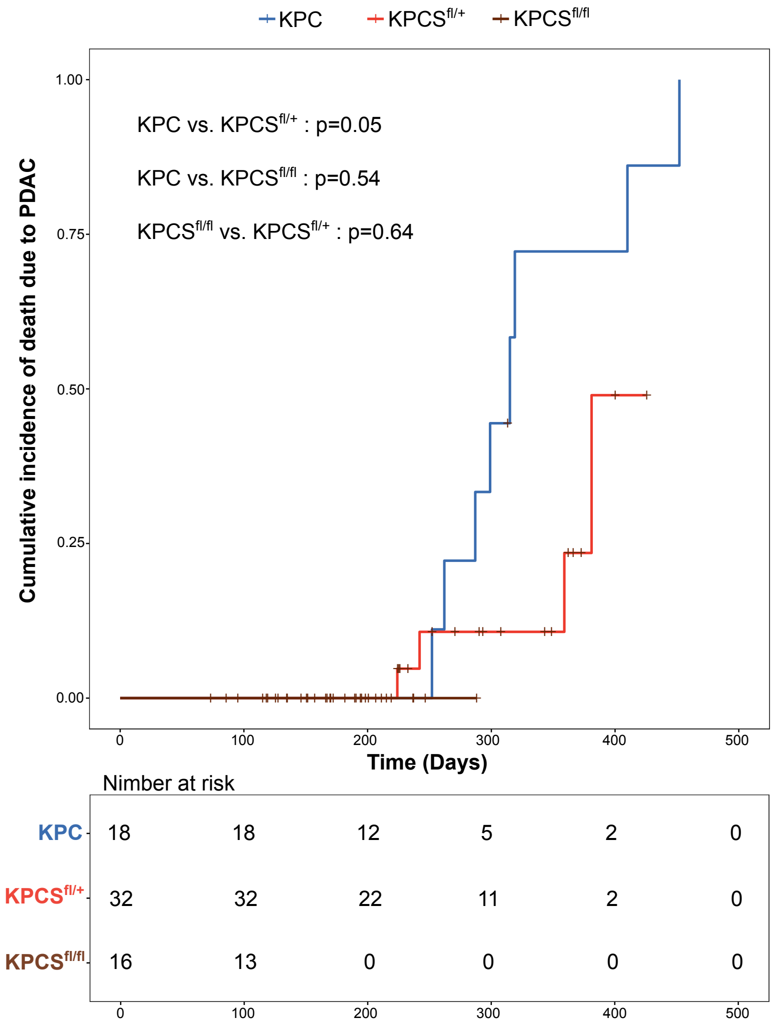

Supplementary Figure 2 continued

E

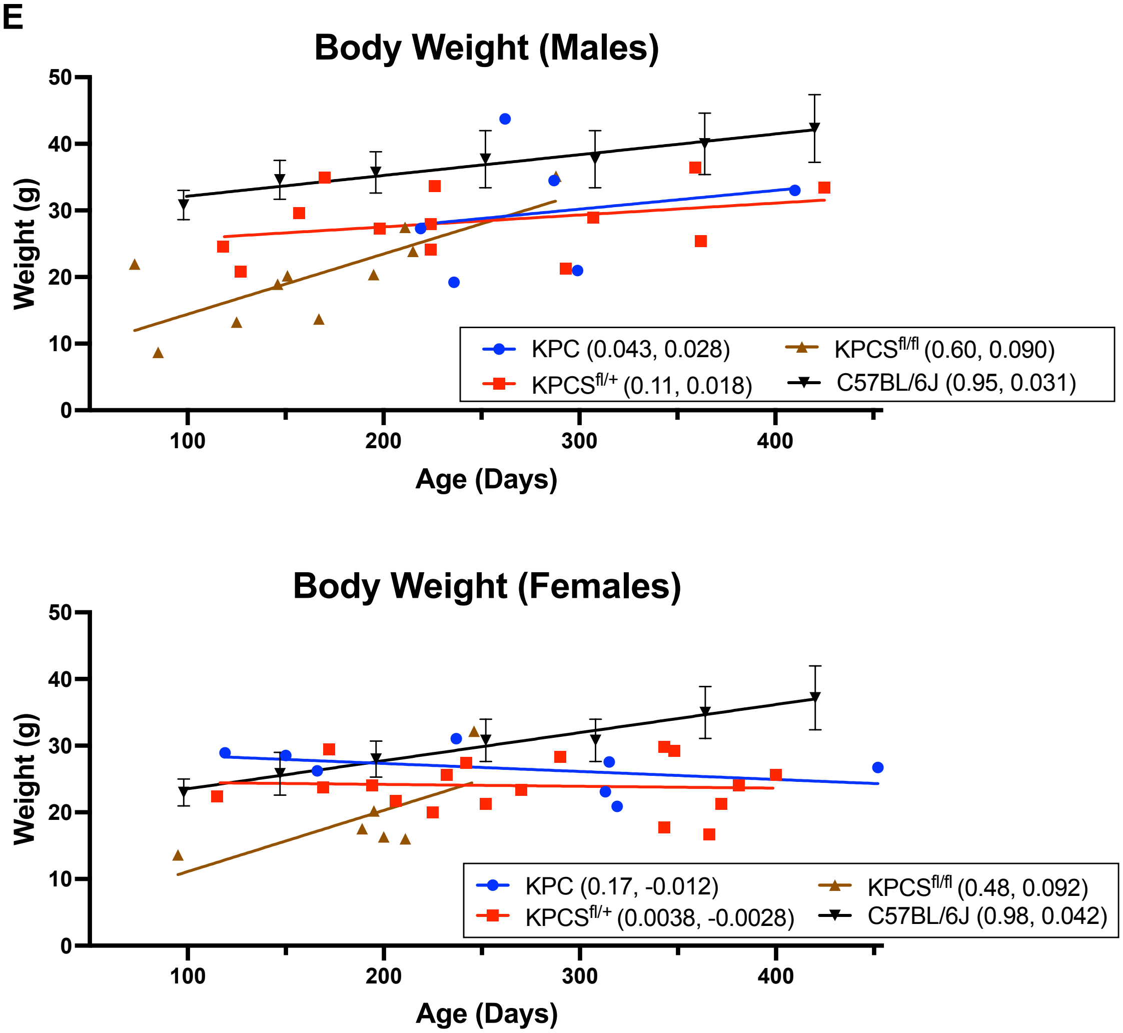

FIGURE S2 – Mouse genotyping methods and survival study data.

A) Representative genotyping of non-pancreatic tissue (tail or ear punch) of all mouse cohorts for identification purposes. *FAIBP* is fatty acid intestinal binding protein, an internal control. **B**) Representative genotyping of pancreas and liver tissue to identify specific genetic recombination events in *Kras*, *Trp53*, and *Scap*. PCR bands were not observed when the amplicons were too large to be amplified using the defined extension time (25 seconds). In the schematic of *Scap* recombination, exon numbers were modified based on updated NCBI RefSeq database. It was originally described that *loxP* sites are located upstream of exon 1 and in intron 1 (24). **C)** Swimmer plot showing lifespan of subjects in study and whether death was due to PDAC. Lifespan in days was calculated using date of birth and date of death. **D)** Figure shows the cumulative incidence curves of death due to PDAC for each genotype group. For this analysis, we considered death due to other causes as a censoring event. Log rank test was used for pairwise comparisons. **E)** Weight and age at time of death from Table S1 were plotted for each mouse in the study. Values in parentheses are (R^2 value, slope) of linear regression curves for each cohort as determined using Prism 10.2.0. Weight data for male and female C57BL/6J mice were obtain from Jackson Labs (https://www.jax.org/jax-mice-and-services/strain-data-sheet-pages/body-weight-chart-aged-b6; https://www.jax.org/jax-mice-and-services/strain-data-sheet-pages/body-weight-chart-000664#). KPC body weights for all genotypes where reduced compared to healthy C57BL/6J reference data, which is not surprising since we measured weight only at time of death.
