# Supplementary material for: SREBP-Dependent Regulation of Lipid Homeostasis Is Required for Progression and Growth of Pancreatic Ductal Adenocarcinoma
Source: Cancer Res Commun. 2024 Sep 27;4(9):2539–52. doi: 10.1158/2767-9764.CRC-24-0120 (PMC11444119; doi:10.1158/2767-9764.CRC-24-0120)
Supplement: Supplementary Figure 3 — FIGURE S3 – Generation of human PDAC SCAP knockout cell lines [file crc-24-0120_supplementary_figure_3_suppsf3.pdf]

Supplementary Figure 3

Human PDAC cell lines: parental (WT) and *SCAP KO* (KO)

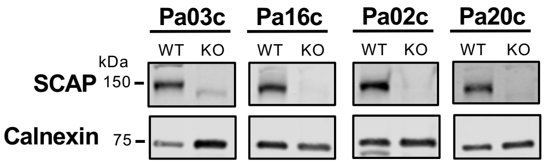

FIGURE S3 – Generation of human PDAC *SCAP* knockout cell lines.

Immunoblot analysis of Pa02c, Pa03c, Pa16c, and Pa20c cells for SCAP. Wildtype (WT) and *SCAP* knockout cells (KO) were cultured in 10% FBS supplemented medium. Membrane-enriched extracts (50 μg) were harvested and probed for SCAP. Calnexin served as a loading control.
