# Supplementary material for: SREBP-Dependent Regulation of Lipid Homeostasis Is Required for Progression and Growth of Pancreatic Ductal Adenocarcinoma
Source: Cancer Res Commun. 2024 Sep 27;4(9):2539–52. doi: 10.1158/2767-9764.CRC-24-0120 (PMC11444119; doi:10.1158/2767-9764.CRC-24-0120)
Supplement: Supplementary Figure 4 — FIGURE S4 – SCAP is required for human PDAC tumor growth in mouse orthotopic xenograft models [file crc-24-0120_supplementary_figure_4_suppsf4.pdf]

Supplementary Figure 4

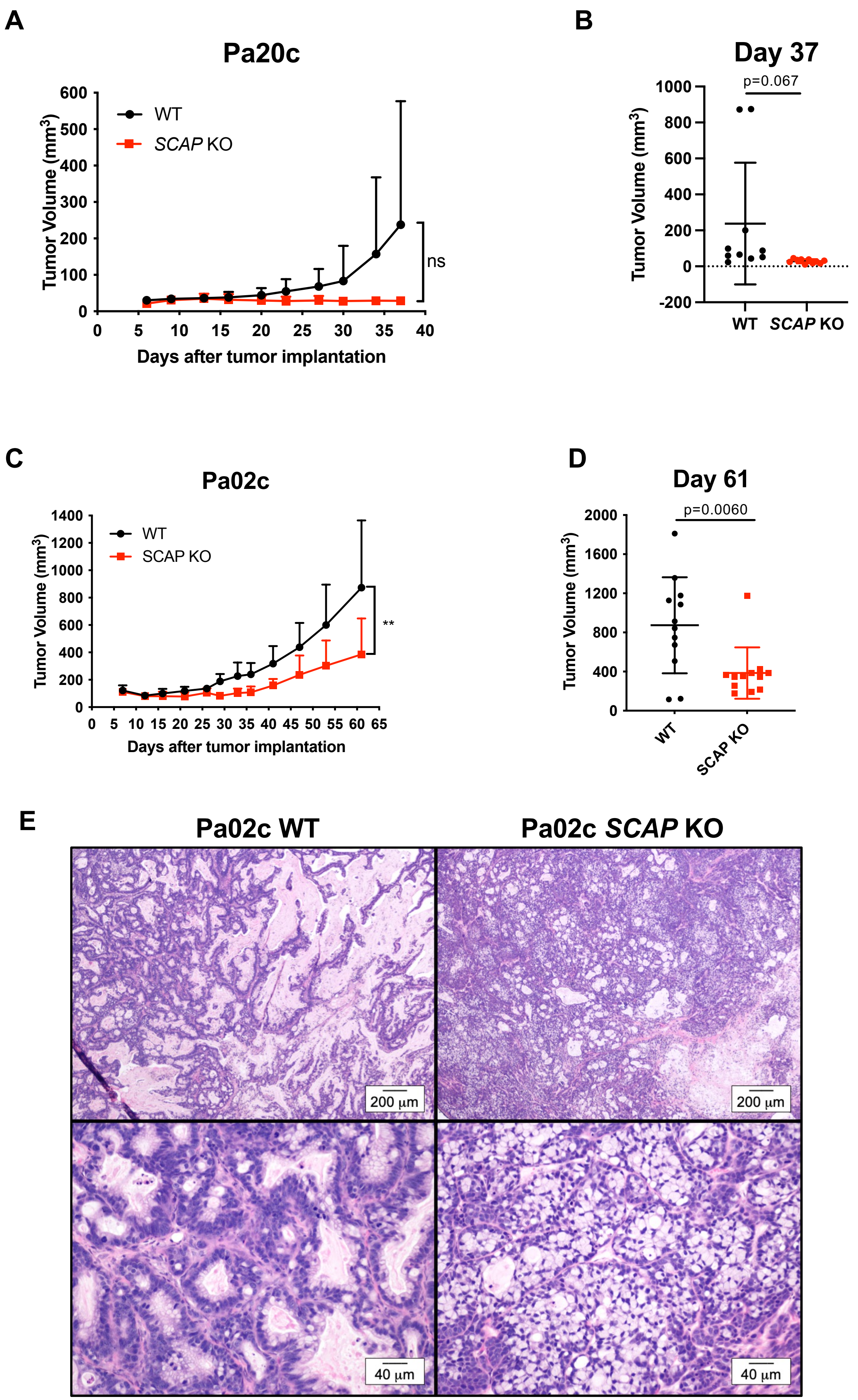

FIGURE S4 – *SCAP* is required for human PDAC tumor growth in mouse orthotopic xenograft models.

A) Nude mice were subcutaneously injected with $1x10^6$ Pa20c cells in both flanks (two tumors per mouse). Once visible, tumors were measured, and volume calculated. Each group contained 5 mice. Data represent mean ± SD (ns, not significant, student's t-test). **B)** Individual tumor volumes at day 37, n=10 tumors per group. **C)** Nude mice were subcutaneously injected with $1x10^6$ Pa02c cells in both flanks (two tumors per mouse). Once visible, tumors were measured, and volume calculated. Each group contained 6 mice. Error bar denotes standard deviation. Statistical significance was determined using student's t-test. (**, $p<0.01$). **D)** Individual tumor volumes at day 61. Error bar denotes standard deviation. Statistical significance was determined using student's t-test. **E)** Representative hematoxylin and eosin (H&E) stained sections of formalin fixed tumor tissues from the mice in **A** and **C** showing a low magnification (4X) image (upper row) and a high magnification (20X) image (bottom row) of tumor sections.
