# Supplementary material for: SREBP-Dependent Regulation of Lipid Homeostasis Is Required for Progression and Growth of Pancreatic Ductal Adenocarcinoma
Source: Cancer Res Commun. 2024 Sep 27;4(9):2539–52. doi: 10.1158/2767-9764.CRC-24-0120 (PMC11444119; doi:10.1158/2767-9764.CRC-24-0120)
Supplement: Supplementary Figure 5 — FIGURE S5 – SCAP is required for PDAC cell growth and survival in low serum conditions. [file crc-24-0120_supplementary_figure_5_suppsf5.pdf]

Supplementary Figure 5

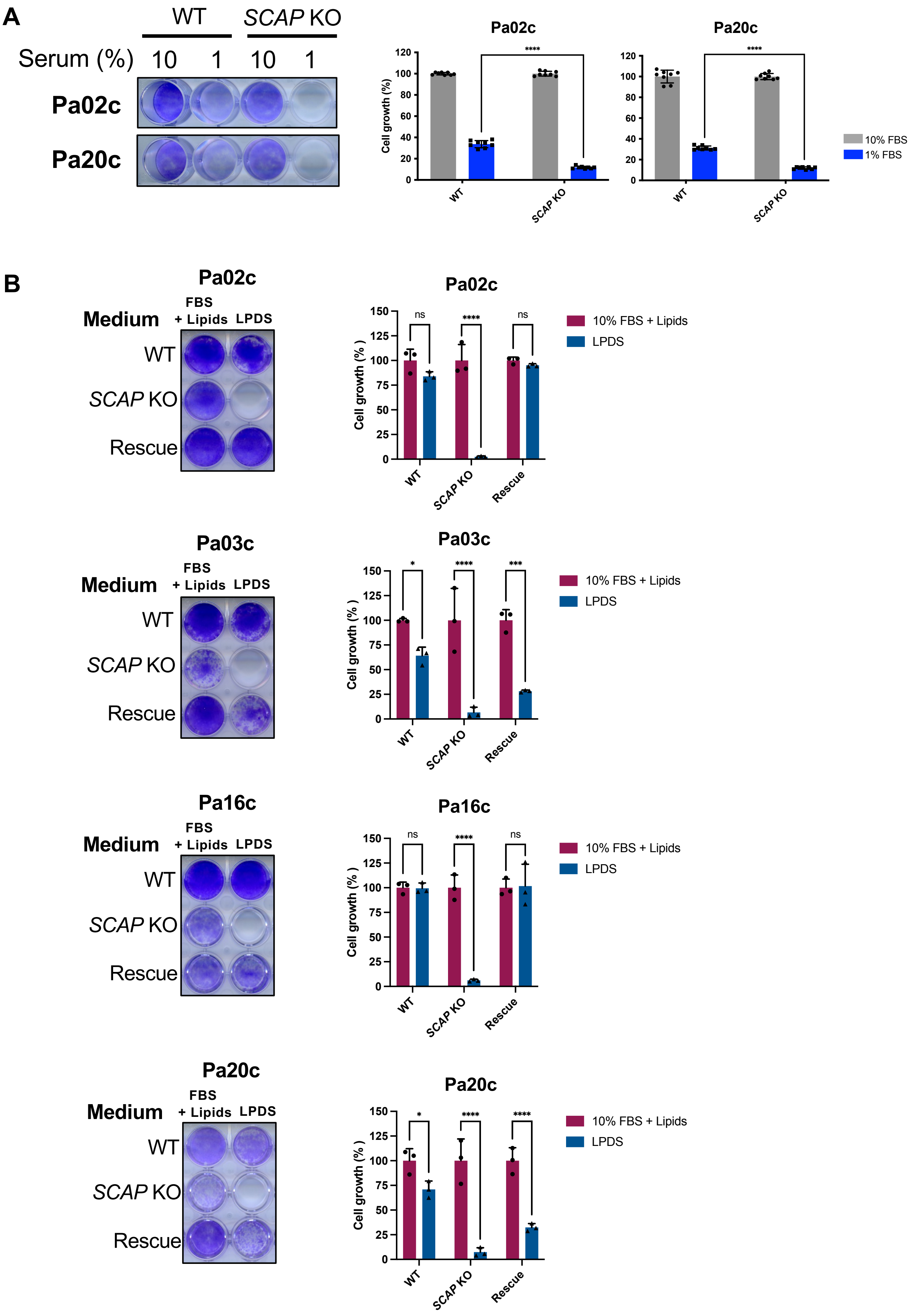

FIGURE S5 – *SCAP* is required for PDAC cell growth and survival in low serum conditions.

A) Cell growth assay of Pa02c and Pa20c cell lines. WT and *SCAP* knockout cells were cultured in either 10% FBS or 1% FBS for 7 days. Plates were stained with crystal violet as a measure of cell proliferation. Quantification of crystal violet staining is shown (n = 3 per group). For each cell line, growth was normalized to the 10% FBS condition. Statistical significance was determined using two-way ANOVA and Tukey's test. P values are indicated: < 0.05 (*); < 0.01 (**); < 0.001 (***); < 0.0001 (****), not significant (ns). Error bar denotes standard deviation. **B)** Cell growth assay of PDAC cell lines. WT, *SCAP* KO, and *SCAP* KO rescued cell lines were cultured in either 10% FBS supplemented with cholesterol (5 µg/mL), mevalonate (1 mM) and oleate-albumin (20 µM) or 10% LPDS with no additions for 7 days. Media were changed every 3 days. Plates were stained with crystal violet as a measure of cell proliferation. Quantification of crystal violet staining is shown (n = 3 per group). For each cell line, growth was normalized to the 10% FBS condition. Statistical significance was determined using two-way ANOVA and Tukey's HSD test. P values are indicated: < 0.05 (*); < 0.01 (**); < 0.001 (***); < 0.0001 (****), not significant (ns). Error bar denotes standard deviation.
