# Supplementary material for: SREBP-Dependent Regulation of Lipid Homeostasis Is Required for Progression and Growth of Pancreatic Ductal Adenocarcinoma
Source: Cancer Res Commun. 2024 Sep 27;4(9):2539–52. doi: 10.1158/2767-9764.CRC-24-0120 (PMC11444119; doi:10.1158/2767-9764.CRC-24-0120)
Supplement: Supplementary Figure 6 — FIGURE S6 – Site-1 protease activation of the SREBP pathway is required for PDAC cell growth in low serum conditions. [file crc-24-0120_supplementary_figure_6_suppsf6.pdf]

Supplementary Figure 6

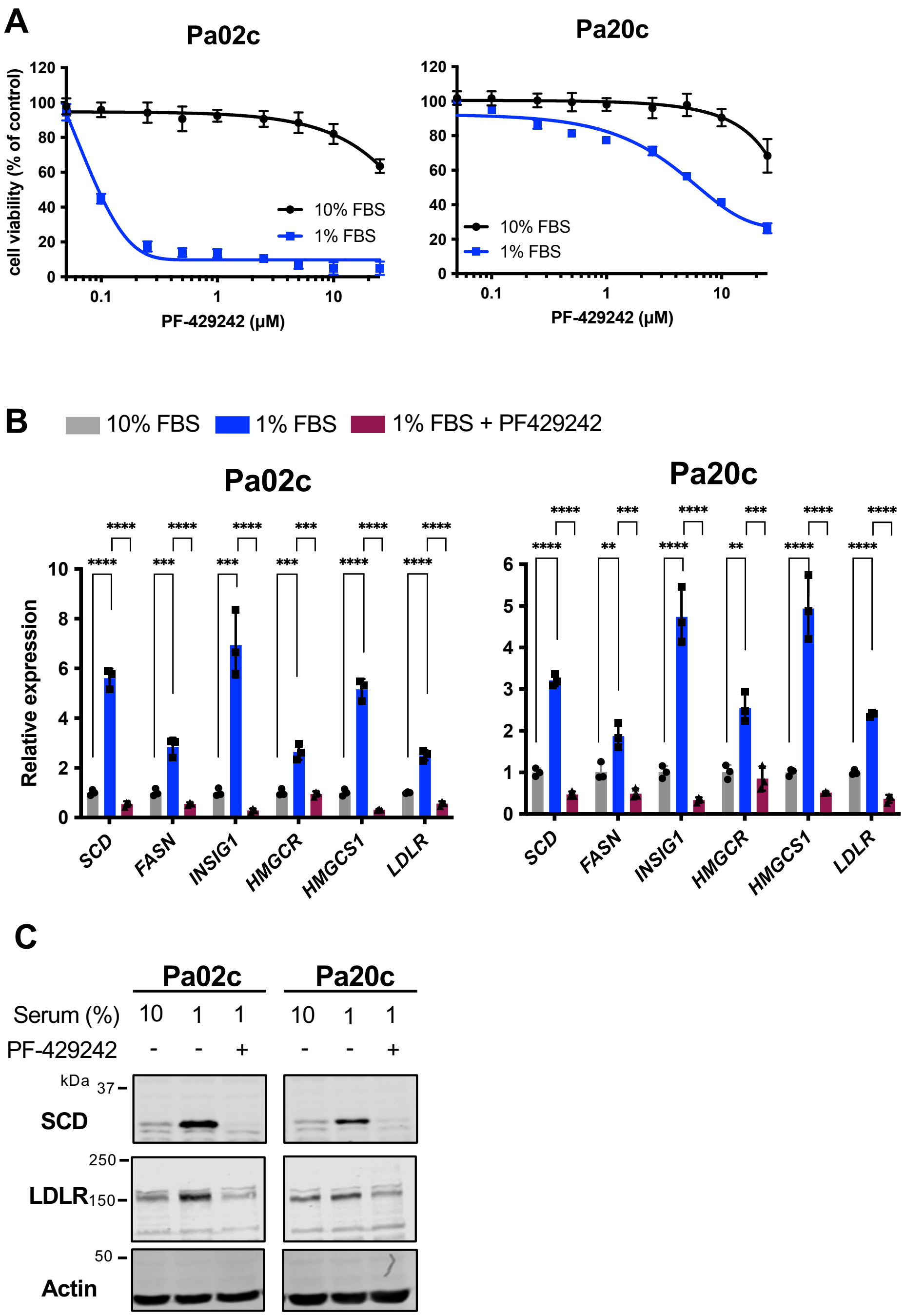

FIGURE S6 – Site-1 protease activation of the SREBP pathway is required for PDAC cell growth in low serum conditions.

A) PDAC cells (Pa02c and Pa20c) were cultured in either 10% or 1% FBS with indicated concentrations of the Site-1 protease inhibitor PF-429242 for 72 hours, and cell growth was determined using a MTS assay with data normalized to 10% FBS untreated cells. Data are representative of 2 biological replicates with 3 technical replicates for each biological replicate. Error bar denotes standard deviation. **B)** Pa02c and Pa20c cells were cultured in 10% FBS, 1% FBS, or 1% FBS with PF-429242 (10 mM) for 16 hours. Quantitative real-time PCR was performed for SREBP-1 (*SCD, FASN, INSIG1*) and SREBP-2 (*HMGCR, HMGCS1, LDLR*) target genes. *GAPDH* served as a control. Data are representative of 2 biological replicates with 3 technical replicates for each biological replicate. Error bar denotes standard deviation. Statistical significance was determined using one-way ANOVA and Tukey's HSD test. P values are indicated: < 0.05 (*); < 0.01 (**); < 0.001 (***); < 0.0001 (****), not significant (ns). **C)** Immunoblot analysis of whole cell lysates from Pa02c and Pa20c cells for SREBP target protein expression. Cells were cultured under the same conditions as in B. Whole cell protein extracts were harvested and probed for either SCD or LDLR. Actin served as a loading control. The figure is representative of 2 biological replicates.
